# Permissive underfeeding, cytokine profiles and outcomes in critically ill patients

**DOI:** 10.1371/journal.pone.0209669

**Published:** 2019-01-07

**Authors:** Yaseen Arabi, Dunia Jawdat, Abderrezak Bouchama, Hani Tamim, Waleed Tamimi, Mohammed Al-Balwi, Hasan M. Al-Dorzi, Musharaf Sadat, Lara Afesh, Mashan L. Abdullah, Walid Mashaqbeh, Maram Sakhija, Mohamed A. Hussein, Adila ElObeid, Abdulaziz Al-Dawood

**Affiliations:** 1 Intensive Care Department, King Saud bin Abdulaziz University for Health Sciences, King Abdullah International Medical Research Center, King Abdulaziz Medical City, Riyadh, Saudi Arabia; 2 Cord Blood Bank, King Abdullah International Medical Research Center, King Saud bin Abdulaziz University for Health Sciences, King Abdulaziz Medical City, Riyadh, Saudi Arabia; 3 Department of Experimental Medicine, King Abdullah International Medical Research Center, King Saud bin Abdulaziz University for Health Sciences, King Abdulaziz Medical City, Riyadh, Saudi Arabia; 4 Department of Internal Medicine, American University of Beirut Medical Center, Beirut, Lebanon; 5 Department of Clinical Laboratory, King Saud bin Abdulaziz University for Health Sciences, King Abdullah International Medical Research Center, King Abdulaziz Medical City, Riyadh, Saudi Arabia; 6 Department of Biostatistics and Bioinformatics, King Abdullah International Medical Research Center, King Saud bin Abdulaziz University for Health Sciences, King Abdulaziz Medical City, Riyadh, Saudi Arabia; 7 Department of Biobank, King Abdullah International Medical Research Center, King Saud bin Abdulaziz University for Health Sciences, King Abdulaziz Medical City, Riyadh, Saudi Arabia; University of Notre Dame Australia, AUSTRALIA

## Abstract

**Background:**

During critical illness in humans, the effects of caloric restriction on the inflammatory response are not well understood. The aim of this study is to examine the associations of caloric restriction, inflammatory response profiles and outcomes in critically ill patients.

**Methods:**

This is a sub-study of the PermiT trial (Permissive Underfeeding or Standard Enteral Feeding in Critically Ill Adults Trial- ISRCTN68144998). Serum samples were collected on study days 1, 3, 5, 7 and 14 and analyzed for a panel of 29 cytokines. We used principal component analysis to convert possibly correlated variables (cytokine levels) into a limited number of linearly uncorrelated variables (principal components). We constructed repeated measures mixed linear models to assess whether permissive underfeeding compared to standard feeding was associated with difference cytokine levels over time.

**Results:**

A total of 72 critically ill patients were enrolled in this study (permissive underfeeding n = 36 and standard feeding n = 36). Principal component analysis identified 6 components that were responsible for 78% of the total variance. When adjusted to principal components, permissive underfeeding was not associated with 90-day mortality (adjusted odds ratio 1.75, 95% confidence interval 0.44, 6.95, p = 0.43) or with incident renal replacement therapy. The cytokines did not differ with time between permissive underfeeding and standard feeding groups.

**Conclusions:**

The association of permissive underfeeding compared to standard feeding with mortality was not influenced by the inflammatory profile. Permissive underfeeding compared to standard feeding was not associated with differences in the serum levels of cytokines in critically ill patients.

## Introduction

The possible effects of caloric and protein intake on inflammatory response have been increasingly recognized. Caloric restriction has been shown to attenuate the inflammatory response associated with diabetes, obesity, cancer, cardiovascular disease and ageing.[1–3] During critical illness, which is typically characterized by an intense pro-inflammatory response, the effects of caloric intake on inflammation are less certain. Animals subjected to caloric restriction mounted less elevation of pro-inflammatory cytokines.[4, 5] During critical illness in humans, the effects of caloric restriction on the inflammatory response are not well understood.

The objective of this sub-study of the PermiT (Permissive Underfeeding versus Target Enteral Feeding in Adult Critically Ill Patients Trial)[6] is to examine the associations of caloric restriction, inflammatory response profiles and outcomes in critically ill patients. We hypothesized that permissive underfeeding attenuates the pro-inflammatory response and that the association of caloric intake with clinical outcomes may differ according to patient inflammatory profile. Some of these findings have been previously presented and published as an abstract.[7]

## Materials and methods

### Study population

This is a sub-study of the PermiT trial, in which critically ill patients were randomized to permissive underfeeding (40–60% of calculated caloric requirements) or standard feeding (70–100%) for up to 14 days while maintaining similar protein intake in both groups.[8] Eligibility criteria and interventions are detailed in the publication of the main trial.[6, 8] The trial found no difference in the primary endpoint of 90-day mortality between the permissive and standard feeding groups [relative risk 0.94, 95% confidence interval (CI) 0.76, 1.16, p = 0.58]. In this a priori sub-study, consecutive patients enrolled in PermiT trial at King Abdulaziz Medical City, Riyadh, Saudi Arabia between September 2012 and September 2014 and expected to stay for ≥14 days as judged by the treating intensivist were approached for informed consent for participation in this study. The study was approved by the Institutional Board Review of the Ministry of the National Guard Health Affairs, Riyadh, Saudi Arabia and all clinical investigations were conducted according to the principles expressed in the Declaration of Helsinki.

### Nutrition

The dietician calculated caloric requirement based on predefined formulae using the Penn State equation for mechanically ventilated patients with BMI <30 kg/m^2^ and the Ireton-Jones equation for mechanically ventilated patients with BMI ≥30 kg/m^2^ and for spontaneously breathing patients[6] and then selected the tube feeding formula depending on the patient condition, usually after discussion with the treating ICU team. Protein target was calculated at 1.2 to 1.5 g g/kg according to the clinical practice guidelines.[9] To achieve similar protein delivery to both feeding groups, additional protein (Resource Beneprotein, Nestle Healthcare) was provided if needed.

### Clinical data

We documented demographics and nutritional intervention data and clinical outcomes including mortality (at ICU and hospital discharge, 90 days, 28 days, 180 days), incident renal replacement therapy, mechanical ventilation duration, ICU and hospital lengths of stay (LOS), and ICU-associated infections during the ICU stay.

### Blood sampling and measurements of cytokines

Blood samples were collected on days 1, 3, 5, 7 and 14 of enrollment in the PermiT trial. Day 1 was the day of randomization, which was within 48 hours of ICU admission. Serum was prepared from the clotted blood samples by centrifugation for 10 minutes at 1000 x g at 4°C, then was stored at −80°C prior to assay. A total of 29 cytokines were measured in duplicates using Milliplex panel (human cytokine/chemokine magnetic bead panel, kit catalog code HCYTMAG-60K-PX29, Merck Millipore, Darmstadt, Germany) with the Luminex 3D platform (Luminex, Austin, TX), according to the manufacturer instructions. The cytokine panel included epidermal growth factor (EGF), eotaxin, granulocyte-colony stimulating factor (G-CSF) and granulocyte-macrophage colony-stimulating factor (GM-CSF), interferon (IFN)-α2 and IFN-γ, interleukin (IL)-1α, IL-1β, IL-1ra, IL-2, IL-3, IL-4, IL-5, IL-6, IL-7, IL-8, IL-10, IL-12 (p40), IL-12 (p70), IL-13, IL-15, IL-17A, inducible protein-10 (IP-10), monocyte chemo-attractant protein (MCP)-1, macrophage inflammatory protein (MIP)-1α, MIP-1β, tumor necrosis factor (TNF)-α, TNF-β, and vascular endothelial growth factor (VEGF).

### Statistical analysis

Due to the exploratory nature of the study, there was no sample size calculation performed. Statistical analyses were performed using SAS version 9.2 (SAS Institute, Cary, NC). We used principal component analysis (PCA) as a data reduction technique to capture the maximum variation patterns in the 29 cytokine levels measured at day one in few linearly independent components.[10] PCA summarizes variation in data without any prior assumptions about whether the samples come from different treatment groups or have phenotypic differences, thus avoids introducing inherent bias.[10] PCA was carried out on the correlation matrix of the 29 cytokine measures at baseline using the “PRINCOMP” procedure of SAS. The analysis generated principal components with a set of eigenvalues and eigenvectors. Eigenvalues measures the magnitude of the variance captured by each principal component. We have sorted the principal components so that the first principal component accounted for the largest possible variance in the data (reflected by the highest eigenvalue), and each following component accounted for as much of the remaining variance as possible. We have retained the top 6 principal components using the scree plot method. The eigenvectors represent a set of weights that describe the loading of each cytokine on the extracted principal components.

We compared baseline characteristics, interventions and outcomes among patients in the permissive underfeeding and standard feeding. We reported categorical variables as frequencies with percentages and continuous variables as medians with quartiles 1 and 3 (Q1, Q3). We compared categorical variables using the chi-square or Fisher’s exact test, as appropriate and continuous variables using Wilcoxon Mann Whitney test. We assessed the association of permissive underfeeding compared to standard feeding with 90-day mortality and incident renal replacement therapy using logistic regression analysis adjusting for the resulting principal components. To assess whether permissive underfeeding compared to standard feeding was associated with difference cytokine levels over time, we constructed repeated measures mixed linear models. A two-tailed P value <0.05 was considered statistically significant.

### Ethics approval and consent to participate

The study was approved by the National Guard Health Affairs Institutional Review Board (IRB), Riyadh, Saudi Arabia. Informed consent was obtained from subjects enrolled in this study.

## Results

A total of 72 patients were enrolled in this sub-study with 36 patients receiving permissive underfeeding and 36 standard feeding (S1 Fig). Their baseline characteristics are presented in Table 1. Patients in the permissive underfeeding group received less calories per day for the study period (up to 14 days) compared with the standard feeding group as per the study criteria [911.4 kcal (789.1, 1072.5) vs. 1137.3 kcal (819.2, 1639.0), p = 0.006]. Other aspects of interventions and co-interventions are reported in Table 2. Clinical outcomes, including mortality (at ICU and hospital discharge, 90 days, 28 days, 180 days), incident renal replacement therapy, mechanical ventilation duration, ICU and hospital lengths of stay (LOS), and ICU-associated infections during the ICU stay, were not different between the two groups (Table 3).

**Table 1:** Baseline characteristics of patients in the permissive underfeeding and standard feeding.

Variable	Permissive underfeeding N = 36	Standard feeding N = 36	P value
**Age–**(yr), median (Q1, Q3)	46.8 (24.1, 70.4)	53.5 (33.0, 71.3)	0.16
**Female sex–**no. (%)	9 (25.0)	12 (33.3)	0.44
**Height–**(cm), median (Q1, Q3)	168 (162, 170)	165 (155, 170)	0.57
**Weight**–(kg), median (Q1, Q3)	74.5 (57.0, 86.0)	72.5 (60.0, 95.0)	0.39
**BMI–**(kg/m^2^), median (Q1, Q3)	26.1 (22.0, 31.2)	29.3 (23.0, 35.7)	0.18
**BMI** ≥ 30 kg/m^2^, no. (%)	11 (30.6)	15 (41.7)	0.33
**Diabetes**, no. (%)	19 (52.8)	19 (52.8)	1.00
**Inclusion blood glucose**–(mmol/L), median (Q1, Q3)	10.9 (8.3, 13.6)	8.7 (6.7, 12.5)	0.12
**Admission category**, no. (%)			
Medical	19 (52.8)	21 (58.3)	0.88
Surgical	4 (11.1)	4 (11.1)	
Post-operative trauma	13 (36.1)	11 (30.6)	
**Renal replacement therapy**, no. (%)	1 (2.8)	1 (2.8)	1.00
**APACHE II**–median (Q1, Q3)	21 (16, 25)	21 (12, 27)	0.92
**Mechanical ventilation**, no. (%)	33 (91.7)	35 (97.2)	0.30
**Severe sepsis at admission**, no. (%)	7 (19.4)	11 (30.6)	0.27
**Vasopressor**, no. (%)	23 (63.9)	25 (69.4)	0.67
**Hemoglobin–**(g/L), median (Q1, Q3)	109 (86, 126)	111 (97, 131)	0.38
**INR–**median (Q1, Q3)	1.1 (1.0, 1.3)	1.1 (1.0, 1.3)	0.88
**SOFA Score on Day 1 –**median (Q1, Q3)	10 (9, 12)	10 (8, 13)	0.58
**PaO**_**2**_**/FiO**_**2**_ **ratio**, median (Q1, Q3)	150.0 (117.0, 269.0)	125.5 (73.5, 242.0)	0.44
**Platelets**–(x 10^9^/L), median (Q1, Q3)	186.0 (141.5, 277.5)	187.0 (137.5, 243.0)	0.55
**Bilirubin–**(μmol/L), median (Q1, Q3)	11.7 (8.6, 26.9)	17.1 (9.7, 27.9)	0.30
**SOFA Hypotension score**–median (Q1, Q3)	3 (3, 4)	4 (1, 4)	0.23
**GCS**–median (Q1, Q3)	3 (3, 6)	3 (3, 6)	0.54
**Creatinine–**(μmol/L), median (Q1, Q3)	97.5 (73.0, 118.0)	84.5 (67.0, 121.0)	0.49
**C-reactive Protein–**(mg/L), median (Q1, Q3))	120.5 (60.7, 190.5)	118.1 (55.4, 171.0)	0.77
**24-hour urinary urea nitrogen–**(mmol/L), median (Q1, Q3)	205 (101, 346)	255 (183, 371)	0.21
**Serum lipid profile**–(mmol/L), median (Q1, Q3)			
Cholesterol	2.5 (1.9, 3.0)	2.6 (2.1, 3.3)	0.36
Triglycerides	1.1 (0.7, 1.6)	1.2 (0.9, 1.8)	0.30
HDL	0.6 (0.4, 0.9)	0.6 (0.4, 0.9)	0.76
LDL	1.0 (0.6, 1.4)	1.2 (0.8, 1.7)	0.23
**Albumin–**(g/L), median (Q1, Q3)	29 (25, 32.5)	28 (27, 31)	0.68
**Prealbumin–**(g/L), median (Q1, Q3)	0.1 (0.1, 0.1)	0.1 (0.08, 0.1)	0.60
**Hemoglobin A1c**, median (Q1, Q3)	0.06 (0.05, 0.07)	0.06 (0.05, 0.07)	0.87
**Minute ventilation–**(L), median (Q1, Q3)	9.4 (7.7, 11.3)	8.6 (7.2, 10.7)	0.28
**Maximum temperature**–(^0^C), median (Q1, Q3)	37.2 (36.7, 37.7)	37.0 (36.6, 37.6)	0.58

**BMI**: body mass index; **APACHE II**: Acute Physiology and Chronic Health Evaluation II; **INR**: international normalized ratio; **SOFA**: Sequential Organ Failure Assessment; **GCS**: Glasgow coma scale **PaO2:FiO2 ratio**: the ratio of partial pressure of oxygen to the fraction of inspired oxygen; **HDL**: High density lipoproteins; **LDL**: Low density lipoproteins. The denominators for all percentages is the N for each column. Continuous variables are represented as median (quartile 1 and quartile 3)

**Table 2:** Nutritional and metabolic data in patients in the permissive underfeeding and standard feeding.

Variable	Permissive underfeeding N = 36	Standard feeding N = 36	P value
**Calculated caloric requirement**–(kcal/day), median (Q1, Q3)	1727 (1613, 2030.5)	1876 (1586, 2062)	0.43
**Study caloric target**–(kcal/day), median (Q1, Q3)	1074 (962, 1218.5)	1876 (1586, 2062)	<0.0001
**Daily caloric intake for the intervention duration**–			
No. of kilocalories, median (Q1, Q3)	911.4 (789.1, 1072.5)	1137.3 (819.2, 1639.0)	0.006
Percent of requirement, median (Q1, Q3)	54.1 (46.7, 57.3)	69.8 (47.4, 88.8)	0.004
**Caloric source for the intervention duration**–(kcal /day), median (Q1, Q3)			
Enteral	785.5 (650.0, 1022.5)	1121.7 (731.1, 1507.9)	0.004
Propofol	75.6 (19.1, 129.9)	73.7 (20.1, 147.6)	0.83
Intravenous dextrose	3.6 (0.0, 22.9)	0.0 (0.0, 32.9)	0.27
Total parenteral nutrition	0 (0, 0)	0 (0, 0)	0.33
**Calculated protein requirement–**(g/day), median (Q1, Q3)	78.5 (68, 90)	83.0 (70.5, 96.5)	0.14
**Daily protein intake for the intervention duration,** median (Q1, Q3)			
No. of grams	55.3 (42.8, 67.1)	53.4 (34.0, 66.5)	0.45
Percent of requirement	72.6 (63.0, 84.0)	63.3 (41.6, 83.3)	0.12
Daily average protein intake/kg (g/day)	0.77 (0.66, 0.91)	0.70 (0.40, 0.92)	0.28
**Protein source**–(g/day), median (Q1, Q3)			
Main enteral formula	27.0 (23.4, 37.0)	50.6 (31.3, 64.1)	<0.0001
Supplemental enteral protein	23.4 (19.2, 34.0)	0.0 (0.0, 1.2)	<0.0001
Parenteral protein	0 (0,0)	0 (0, 0)	0.33
**Duration of intervention**–(days), median (Q1, Q3)	11 (7, 14)	13 (5.5, 14.0)	0.86
**Co-interventions during study period**			
**Insulin**			
Use–no. (%)	21 (58.3)	24 (66.7)	0.47
Dose–(units/day), median (Q1, Q3)	4.6 (0.0, 29.0)	6.7 (0.0, 31.8)	0.53
**Blood glucose**–(mmol/L), median (Q1, Q3)	7.7 (6.7, 10.3)	9.0 (6.7, 11.5)	0.35
**Enteral formulae on day 1** ^–^no. (%)			
Disease-non-specific	16 (44.4)	15 (41.7)	0.66
Disease-specific	20 (55.6)	21 (58.3)	

**Disease-non-specific formula**: Osmolite, Jevity, Promote, Ensure plus, Resource, Ensure, Resource plus, Jevity (1.2).

**Disease-specific formula**: Glucerna, Nutric hepatic, Nepro, Pulmocare, Novasource Renal, Peptamen (1.0), Peptamen (1.2), Suplena, Oxepa.

**Table 3:** Outcome data in patients in the permissive underfeeding and standard feeding.

Variable	Permissive underfeeding N = 36	Standard feeding N = 36	P value
**Death by 28 days**–no. (%)	5 (13.9)	4 (11.1)	0.92
**Death by 90 days**–no. (%)	7 (19.4)	5 (13.9)	0.53
**Death by 180 days–**no. (%)	7 (19.4)	7 (19.4)	1.00
**Death in the ICU**–no. (%)	4 (11.1)	3 (8.3)	0.69
**Death in the hospital–**no. (%)	5 (13.9)	5 (13.9)	1.00
**New renal replacement therapy**–no. (%)	3 (6.1)	9 (25.7)	0.06
**ICU-associated infections–**no. (%)	19 (52.8)	19 (52.8)	1.00
**ICU length of stay–**(days), median (Q1, Q3)	16 (10, 21)	15 (10, 21)	0.76
**Hospital length of stay**–(days), median (Q1, Q3)	33 (17, 72)	40 (22, 86)	0.23
**Duration of mechanical ventilation**–(days), median (Q1, Q3)	10 (7, 16)	11 (5, 17)	0.93

ICU: intensive care unit

### Principal component analysis

PCA reduced the original 29 cytokines to 6 components that could explain 78% of the total variance. The principal component 1 explained 42% of the total variance, while the other principal components 2–6 were responsible for 12%, 10%, 6%, 4% and 4% respectively (S1 Table). S2 Table represents the loading of each of the 29 cytokines on the retained 6 principal components. GM-CSF, IFN-α2, IL-1β, IL-2, IL-3, IL-4, IL-7, IL-12 (p40), IL-12 (p70), IL-13, IL-15, IL-17A,TNF-α and TNF-β had a loading weight value greater than 0.2 on the principal component 1.

### Association of permissive underfeeding with outcomes adjusting for baseline cytokine principal components

When adjusted to principal components, permissive underfeeding was not associated with 90-day mortality (adjusted odds ratio (aOR) 1.97, 95% CI 0.47, 7.89, p = 0.34) or with incident renal replacement therapy (aOR 0.36, 95% CI 0.07, 1.96, p = 0.24, Table 4). None of the principal components were generally associated with 90-day mortality or incident renal replacement therapy.

**Table 4:** The association of permissive underfeeding compared to standard feeding with 90-day mortality and incident renal replacement therapy adjusted for the principal components of the cytokines.

	90-day mortality		Incident renal replacement therapy	
	aOR (95% CI)	P value	aOR (95% CI)	P value
**Permissive underfeeding vs Standard feeding**	1.75 (0.44–6.95)	0.43	0.25 (0.04–1.45)	0.12
**Principal component 1**	1.07 (0.87–1.31)	0.52	4.82 (0.91–25.69)	0.07
**Principal component 2**	1.14 (0.85–1.53)	0.39	0.71 (0.37–1.37)	0.31
**Principal component 3**	1.35 (0.84–2.18)	0.22	2.37 (0.43–12.95)	0.32
**Principal component 4**	1.14 (0.72–1.82)	0.58	1.02 (0.61–1.72)	0.93
**Principal component 5**	0.76 (0.43–1.33)	0.33	0.70 (0.41–1.22)	0.21
**Principal component 6**	1.17 (0.62–2.23)	0.63	3.08 (1.26–7.50)	0.01

aOR: adjusted odds ratio, CI: confidence interval

### Effect of permissive underfeeding on cytokine levels over time

**Fig 1:**
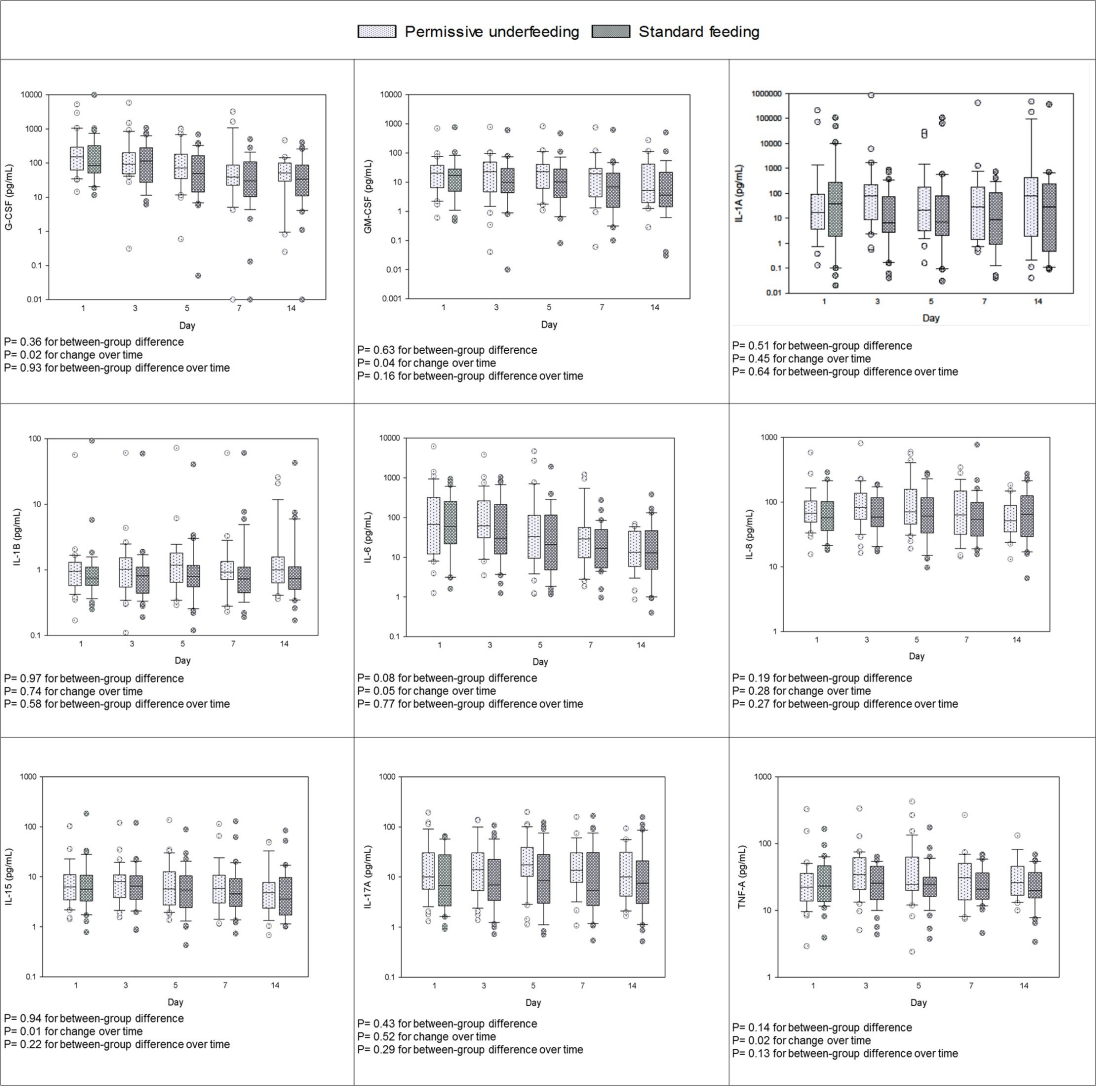
Serial measurements for selected acute pro-inflammatory cytokines in patients randomized to permissive underfeeding and standard feeding groups. The differences between groups, with time and between groups with time (group*time) were tested by repeated measures mixed linear models. Box plots are displayed with medians and quartiles 1 and 3. The error bars refer to 10^th^ and 90^th^ percentiles.

There was no difference in any of the cytokine levels over time between the permissive underfeeding and standard feeding (Fig 1 and S2 Fig).

## Discussion

Our study showed that the association of permissive underfeeding compared to standard feeding (with similar protein intake provided) with mortality and incident renal replacement therapy was not influenced by the inflammatory profile. Cytokine components were not associated with outcomes. Permissive underfeeding compared to standard feeding (with similar protein intake provided) was not associated with any major differences in the inflammatory response in critically ill patients.

Caloric restriction has been associated with increased life span and protection against diabetes, cardiovascular disease and cancer in animals and non-critically ill humans.[11] There is accumulating evidence that caloric restriction may modulate inflammatory response in these settings. A review of the published data suggests that caloric restriction may protect against aging of the immune system, maintain naive T cells, and modify the host response to infection.[12] A randomized controlled trial in 316 older obese adults showed that diet induced weight-loss led to significant reduction in C-reactive protein (CRP), IL-6, and soluble TNF-α receptor 1.[2] A study evaluated a 15-week lifestyle intervention (hypocaloric diet and daily exercise) in 27 severely obese patients and found that the intervention was associated with significant decrease in IL-6, IL-8, and TNF-α.[13] In a pre-post study, 8-week hypocaloric diet in 41 obese subjects led to reduction in CRP, complement C3 and TNF-α levels compared to baseline.[14]

In animal models of critical illness, caloric intake has been shown to influence the inflammatory response. Mice subjected to 40% energy-restriction for 4 weeks, showed less elevation of IL-1β, IL-6 and TNF-α after lipopolysaccharide injection compared with mice in the control group.[4] In another prospective controlled laboratory experiment in mice, 40% dietary restriction for 3 weeks followed by induction of abdominal sepsis or endotoxemia was associated with significantly reduced visceral fat-derived messenger RNA expression of IL-6, thrombospondin-1, plasminogen activator inhibitor-1, and tissue factor and of serum IL-6 and with improved survival.[5]

In critically ill patients, enteral nutrition (EN) may modulate inflammation through several potential mechanisms including affecting the gastrointestinal microbiome,[15] maintaining the intestinal mucosal barrier function[16] and restoring gut immunity.[17] However, the effect of EN on cytokines has not been universally demonstrated. A randomized controlled trial of EN versus parenteral nutrition in 50 patients with severe pancreatitis showed that EN was not associated with changes in IL-6, IL-8 or CRP.[18] A study randomized 20 ICU patients to either receive EN alone or similar EN feeding with supplemental parenteral nutrition to increase caloric intake for 7 days and found that IL-1, IL-6 and TNF-α measured on days of 0, 3 and 7 did not differ between the two groups.[19]

Data on the effect of caloric intake on cytokines in critically ill patients are scarce. In 103 mechanically ventilated patients, markers of inflammation (TNF-α, IL-1β, IFN-γ, IL-6, IL-8, IL-10, IL-12) were not different at day 6 between patients who were randomized to receive trophic feeds (15±11% of target calories) compared to the full calorie group (75 ±39%).[20, 21] The current study was similar in that both used EN with standard non-immune enhancing formulae. However, it differed in that caloric restriction in our study was moderate but more prolonged (up to 14 days versus up to 6 days) and supplemental protein was provided to the patients in the permissive underfeeding group, so the restriction involved only calories.[6, 20, 21] Nevertheless, the two studies showed no major difference in cytokines with caloric restriction. In addition, our study shows that the association of caloric intake with clinical outcomes was not different even after accounting for cytokine profiles.

Why the effects of caloric restriction on cytokines in obesity and in animal models were not observed in critically ill patients in our study? There may be multiple reasons. First, the population was different: our patients were severely ill with a mean APACHE II score of 21.2±10.1, 68 (94.4%) ventilated and 48 (66.7%) on vasopressors, so data on patients who are less severely ill may not be generalizable to ICU patients. Second, the inflammatory response during critical illness is different from that observed in chronic conditions like obesity: our patients had a predominantly acute pro-inflammatory profile, characteristic of critical illness and may correlate with evolution of acute organ dysfunction.[22–26] This was observed in our study by the predominance of pro-inflammatory cytokines loading on principal component 1. Furthermore in mice the plasma levels of IL-6 were significantly reduced by dietary restriction and correlated with adipose IL-6 messenger RNA levels and fat mass suggesting that the action of dietary restriction on suppressing IL-6 production was mediated by its effect on resident adipose cells.[5] Whether similar effect on adipose cells occurs with moderate short-term caloric restriction, like in our trial, remains unknown. Third, the intensity of the intervention is different: caloric differences in our study were less intense and were for shorter period (14 days) than in many other studies in non-critically ill patients or in animal models. Unlike what has been achieved in animal models but similar to other randomized clinical controlled trials of normocaloric versus hypocaloric enteral nutrition, the permissive underfeeding feeding in our study was not very hypocaloric and the standard feeding did not achieve full caloric target.[27, 28] Although we achieved a significant difference in caloric intake between the two groups, the target caloric intake was not reached in the standard- group, and as such, the differences in caloric intake were moderate. This was most likely due to feeding intolerance and feeding interruptions which are common in critically ill patients. This may have reduced the chance of identifying an effect on cytokine levels. Feeding at 100% or more of the energy target during the acute phase of critical illness may be harmful compared to lower caloric levels that are comparable to what we achieved in both study arms.[29–31] Therefore, the results may not be generalizable to patients being fed at full energy target; and our findings cannot exclude that full feeding during the acute phase of critical illness may elicit an inflammatory response. Fourth, the timing of the intervention is different: in animal studies, animals were “pre-conditioned” with several weeks of caloric restriction before inducing critical illness, while in critically ill patients the nutritional interventions follow the critical illness. Fifth, we provided similar protein intake to both groups. There is accumulating evidence suggesting that meeting protein goals and not caloric goals is associated with improved outcomes.[31–34] Therefore, it is possible that an effect of caloric restriction on cytokines in our study might have been “masked” by different effects of protein. Sixth, given the results of the recent NUTRIREA-2 trial, one may question whether a potential beneficial effect of EN on reducing inflammatory response might have been masked by an effect of EN triggering subclinical intestinal ischemia (resulting in increased cytokine levels) in patients with hemodynamic failure, who constituted two thirds of the study population. In the NUTRIREA-2 trial, ventilated patients who were in shock were randomized to receive early EN or early parenteral nutrition within 24 hours after endotracheal intubation aiming to achieve nutritional goals on day 1.[35] The study found no difference in the primary endpoint of all-cause mortality at 28 days. However, early EN was associated with a 4-fold increase in ischemic bowel and colonic pseudo-obstruction.[35] Sixth, the large variation of cytokine levels in critically ill patients, which is reflected in the wide interquartile ranges for all cytokines might have precluded detecting differences in cytokines with the current sample size. Seventh, circulating cytokines may not reflect local inflammatory processes. For example, extracorporeal blood purification therapies in septic patients have been shown to be effective in clearing inflammatory mediators from the plasma, but there effect on mortality has not been demonstrated.[36] Finally, nutrition might also influence the inflammatory response by modulating autophagy, a pathway that may not be reflected in cytokine profiles.[37]

The study should be interpreted in the light of its strengths and weaknesses. The strengths include that it was sub-study of a randomized controlled trial; therefore, the assignment to permissive and standard feeding was randomized. We tested for 29 cytokines to obtain a comprehensive picture about the inflammatory response. Serial serum samples at 5 time points were obtained. We used PCA as a data reduction technique, so the variance from all 29 cytokines can be accounted for in the multivariate analysis. The study limitations include being single centered study. Because of the large variability in cytokines and the sample size, the study may have been underpowered to detect differences in cytokine levels. Additionally, the differences in calories achieved in the clinical setting of the trial were moderate and it remains unclear whether more profound differences would affect cytokines. The study was also not sufficiently powered to perform subgroup analyses.

## Conclusion

In conclusion, the association of permissive underfeeding compared to standard feeding (with similar protein intake provided) with mortality was not influenced by the inflammatory profile. Permissive underfeeding compared to standard feeding was not associated with differences in the serum levels of cytokines in critically ill patients. Whether delivering more restricted caloric intake (lower than what we have achieved in the permissive underfeeding group) reduces inflammatory response or alternatively whether delivering full caloric intake (higher than what we have achieved in the standard feeding group) elicits an inflammatory response with or without full protein supplementation remains to be studied.

## Supporting information

S1 Fig Flow diagram for patients enrolled in the study. (DOCX)

S2 Fig Serial measurements for different cytokines in patients randomized to permissive underfeeding and standard feeding groups. The differences between groups, with time and between groups with time (group*time) were tested by repeated measures mixed linear models. Box plots are displayed with medians and quartiles 1 and 3. The error bars refer to10th and 90th percentiles.
(DOCX)

S1 Table Retained principal components accounting for 79% of the observed variance in the 29 cytokines measured at day 1. (DOCX)

S2 Table Loading coefficients of the 29 cytokines on the retained six principal components. (DOCX)

S1 File Data set. Excel sheet with data related to the baseline characteristic Table 1, nutritional data Table 2, outcome data Table 3 and data related to the Fig 1.
(XLSX)

## Abbreviations

PCA: Principal component analysis
ICU: Intensive care unit
AOR: Adjusted odds ratio
EN: Enteral nutrition
APACHE II: Acute Physiology and Chronic Health Evaluation
