# Supplementary material for: Permissive underfeeding, cytokine profiles and outcomes in critically ill patients
Source: PLoS One. 2019 Jan 7;14(1):e0209669. doi: 10.1371/journal.pone.0209669 (PMC6322779; doi:10.1371/journal.pone.0209669)
Supplement: S2 Fig — The differences between groups, with time and between groups with time (group*time) were tested by repeated measures mixed linear models. Box plots are displayed with medians and quartiles 1 and 3. The error bars refer to10th and 90th percentiles. (DOCX) [file pone.0209669.s002.docx]

**S 2 Fig**

|  | |
| --- | --- |
| **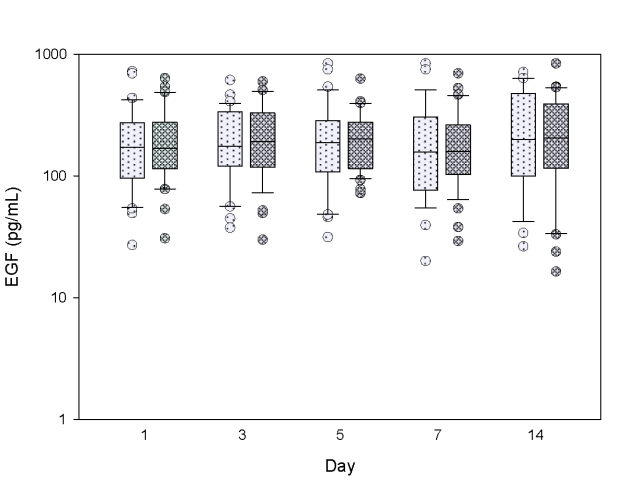**  P= 0.85 for between-group difference  P= 0.11 for change over time  P= 0.76 for between-group difference over time | **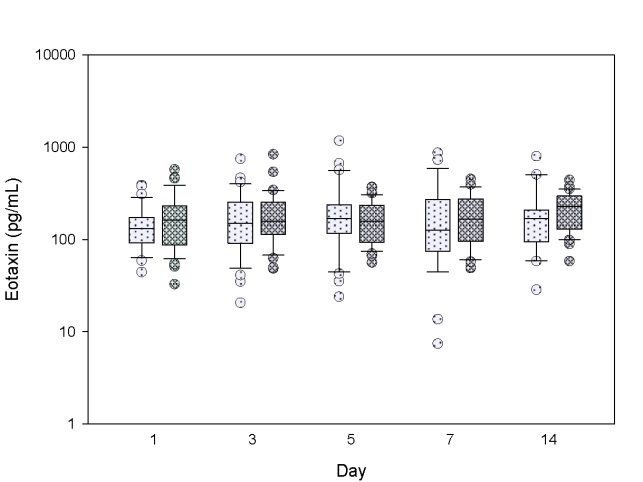**  P= 0.81 for between-group difference  P= 0.02 for change over time  P= 0.05 for between-group difference over time |
| **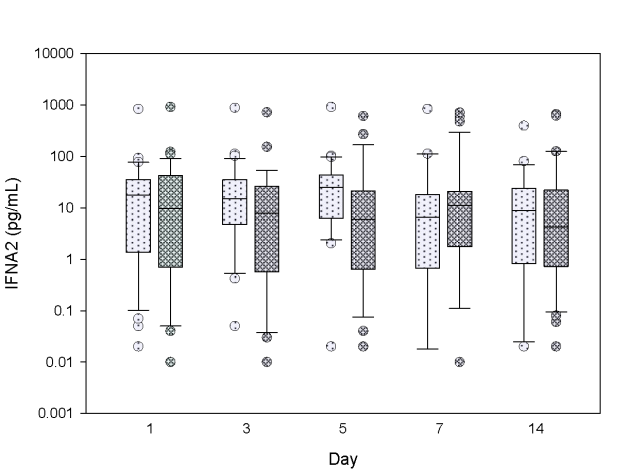**  P= 0.88 for between-group difference  P= 0.63 for change over time  P= 0.12 for between-group difference over time | **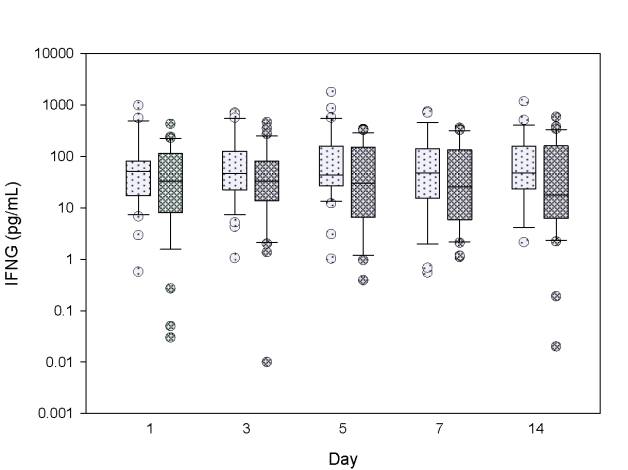**  P= 0.16 for between-group difference  P= 0.79 for change over time  P= 0.75 for between-group difference over time |
| 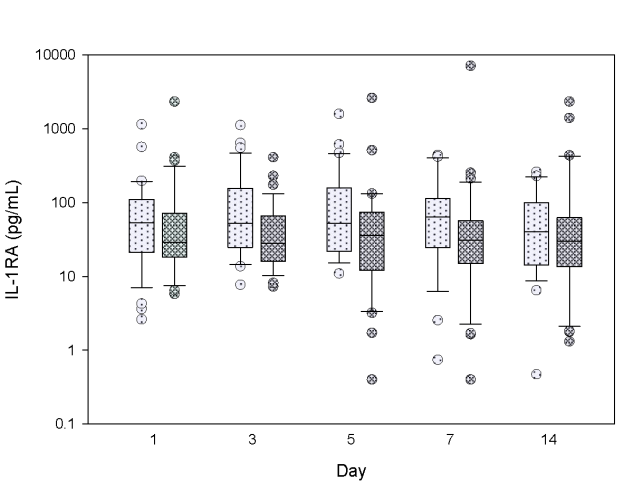  P= 0.66 for between-group difference  P= 0.85 for change over time  P= 0.50 for between-group difference over time | **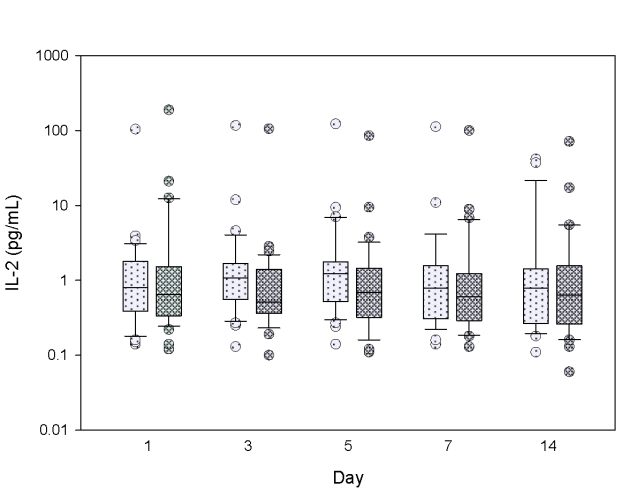**  P= 0.98 for between-group difference  P= 0.36 for change over time  P= 0.28 for between-group difference over time |

|  | |
| --- | --- |
| 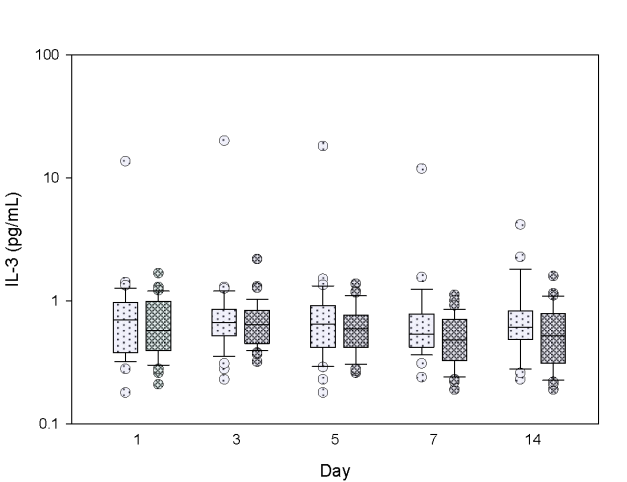  P= 0.32 for between-group difference  P= 0.13 for change over time  P= 0.47 for between-group difference over time | 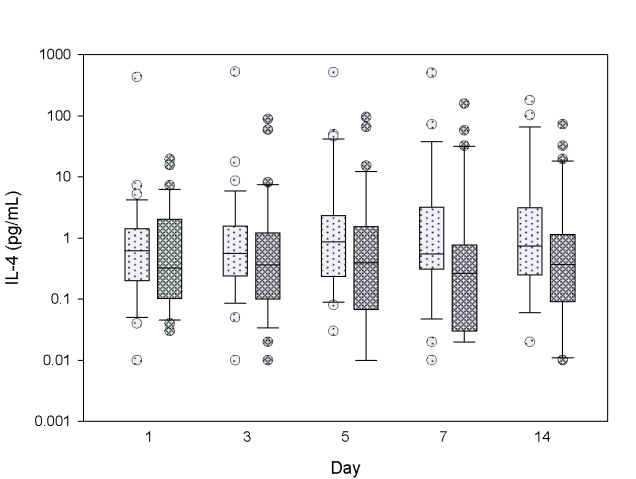  P= 0.44 for between-group difference  P= 0.21 for change over time  P= 0.84 for between-group difference over time |
| 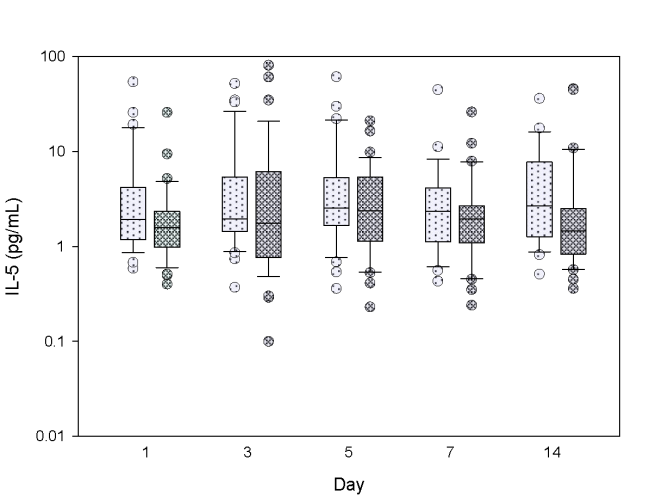  P= 0.51 for between-group difference  P= 0.06 for change over time  P= 0.53 for between-group difference over time | 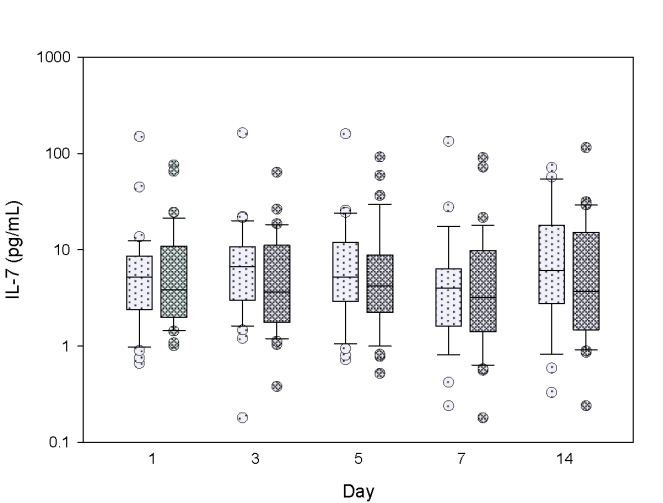  P= 0.66 for between-group difference  P= 0.72 for change over time  P= 0.76 for between-group difference over time |
| 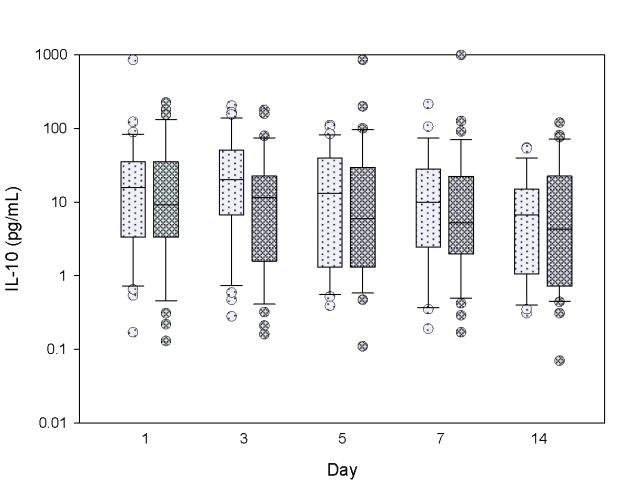  P= 0.86 for between-group difference  P= 0.34 for change over time  P= 0.62 for between-group difference over time | **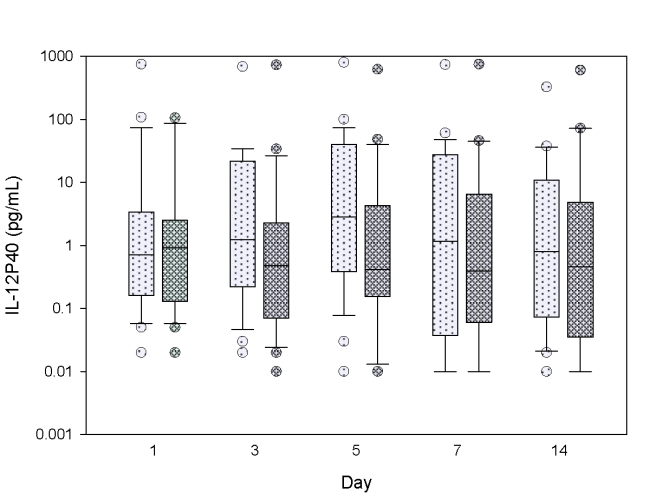**  P= 0.96 for between-group difference  P= 0.11 for change over time  P= 0.14 for between-group difference over time |

|  | |
| --- | --- |
| 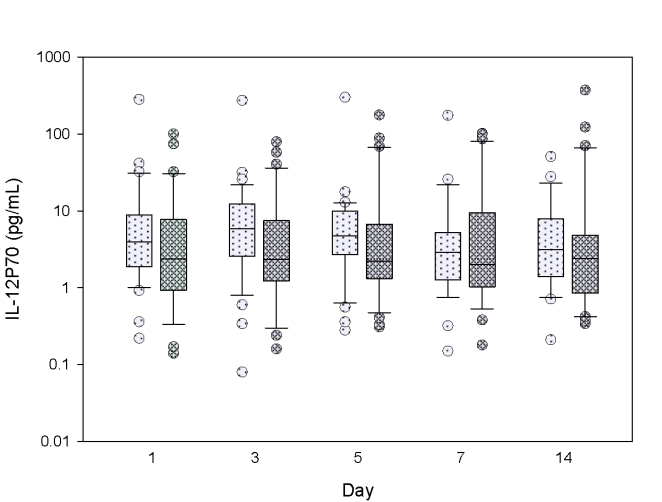  P= 0.71 for between-group difference  P= 0.96 for change over time  P= 0.05 for between-group difference over time | **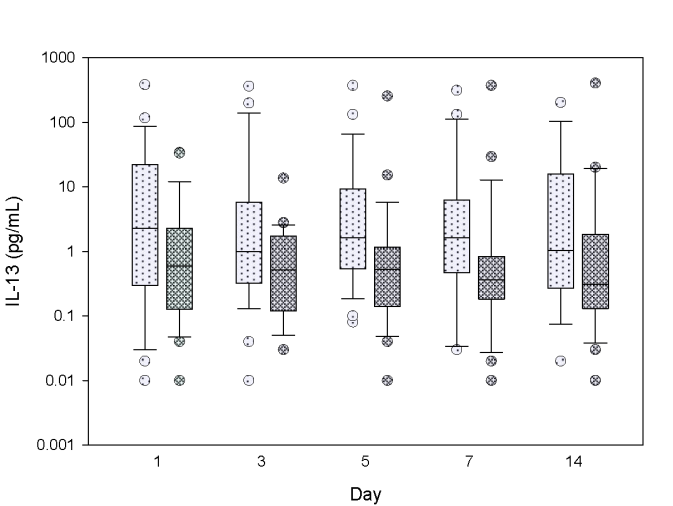**  P= 0.34 for between-group difference  P= 0.99 for change over time  P= 0.27 for between-group difference over time |
| 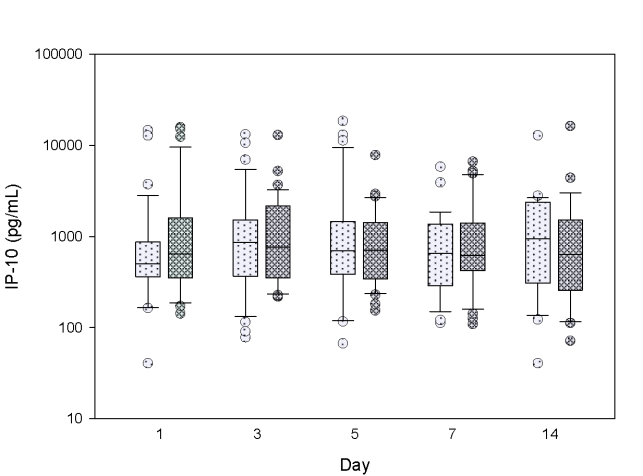  P= 0.79 for between-group difference  P= 0.18 for change over time  P= 0.11 for between-group difference over time | **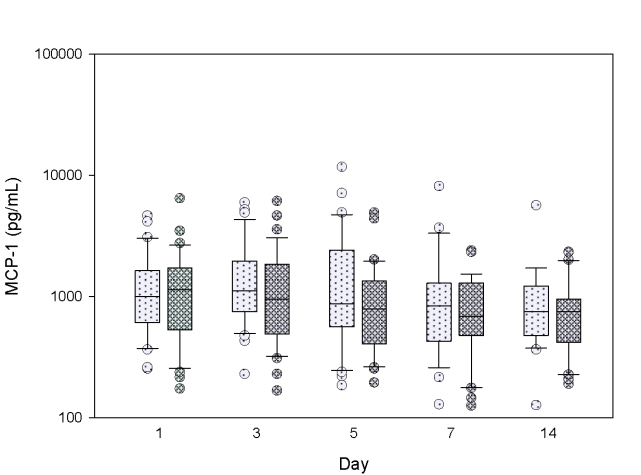**  P= 0.09 for between-group difference  P= 0.02 for change over time  P= 0.26 for between-group difference over time |
| 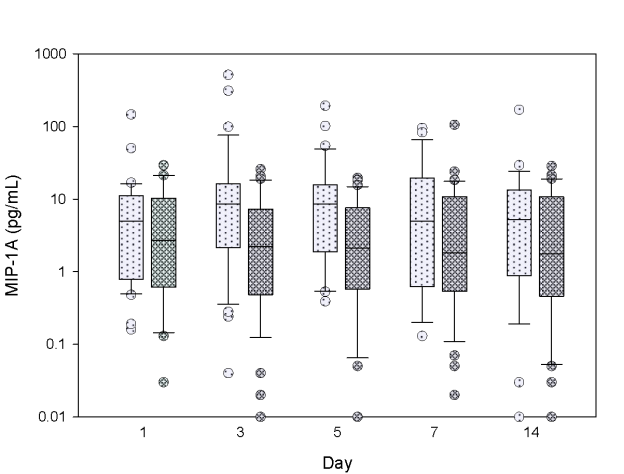  P= 0.07 for between-group difference  P= 0.13 for change over time  P= 0.07 for between-group difference over time | 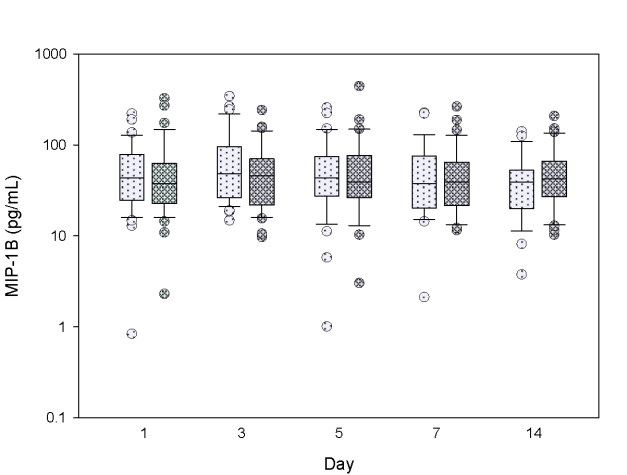  P= 0.90 for between-group difference  P= 0.03 for change over time  P= 0.36 for between-group difference over time |
| 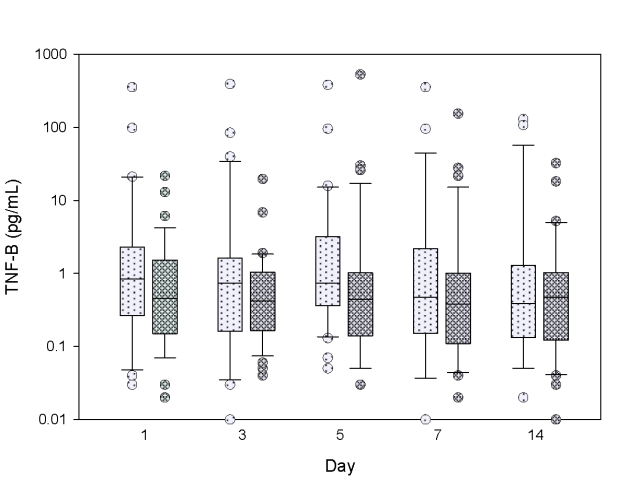P= 0.39 for between-group difference  P= 0.36 for between time  P= 0.58 for between-group difference over time | **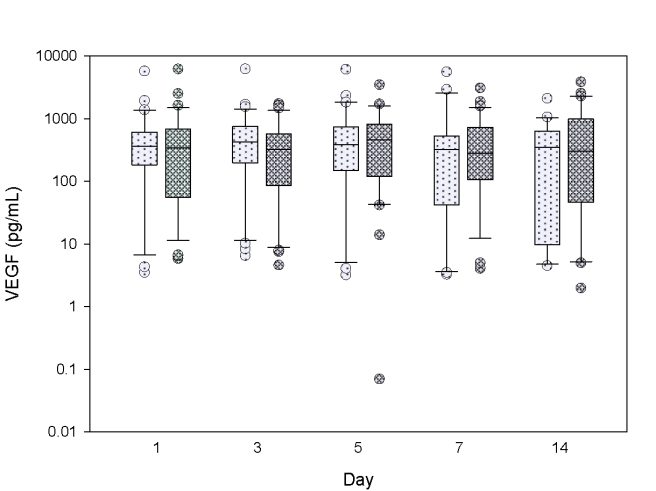**  P= 0.92 for between-group difference  P= 0.60 for change over time  P= 0.02 for between-group difference over time |
