# Supplementary material for: Permissive underfeeding, cytokine profiles and outcomes in critically ill patients
Source: PLoS One. 2019 Jan 7;14(1):e0209669. doi: 10.1371/journal.pone.0209669 (PMC6322779; doi:10.1371/journal.pone.0209669)
Supplement: S1 Table — (DOCX) [file pone.0209669.s003.docx]

**S1 Table:** Retained principal components accounting for 79% of the observed variance in the 29 cytokines measured at day 1.

| **Principal component** | **Eigenvalue** | **Difference** | **Proportion** | **Cumulative** |
| --- | --- | --- | --- | --- |
| 1 | 12.2547231 | 8.8564238 | 0.4226 | 0.4226 |
| 2 | 3.3982993 | 0.4011983 | 0.1172 | 0.5398 |
| 3 | 2.9971010 | 1.3826514 | 0.1033 | 0.6431 |
| 4 | 1.6144496 | 0.3812103 | 0.0557 | 0.6988 |
| 5 | 1.2332393 | 0.0768935 | 0.0425 | 0.7413 |
| 6 | 1.1563458 |  | 0.0399 | 0.7812 |
