# Supplementary material for: Permissive underfeeding, cytokine profiles and outcomes in critically ill patients
Source: PLoS One. 2019 Jan 7;14(1):e0209669. doi: 10.1371/journal.pone.0209669 (PMC6322779; doi:10.1371/journal.pone.0209669)
Supplement: S2 Table — (DOCX) [file pone.0209669.s004.docx]

**S2 Table:** Loading coefficients of the 29 cytokines on the retained six principal components.

|  | **Principal component 1** | **Principal component 2** | **Principal component 3** | **Principal component 4** | **Principal component 5** | **Principal component 6** |
| --- | --- | --- | --- | --- | --- | --- |
| **EGF** | 0.039218 | -0.09917 | **0.241040*** | 0.0447131 | -0.376448 | -0.197511 |
| **EOTAXIN** | 0.090344 | -0.000376 | 0.105245 | -0.280088 | **-0.650681** | -0.030626 |
| **G-CSF** | 0.035813 | 0.189418 | -0.002643 | **0.382151*** | **0.567183*** | 0.016446 |
| **GM-CSF** | **0.260741*** | -0.028273 | **0.214393*** | -0.019956 | 0.023259 | 0.035221 |
| **IFN-α2** | **0.259944*** | 0.049983 | **0.216795*** | -0.001076 | 0.011327 | 0.05952 |
| **IFN-γ** | 0.179766 | -0.041494 | -0.168572 | 0.08598 | -0.056437 | -0.453985 |
| **IL-1α** | -0.028014 | -0.061425 | -0.023547 | -0.187659 | 0.080122 | 0.455033 |
| **IL-1β** | **0.231824*** | -0.081616 | **0.311819*** | -0.030194 | -0.035285 | 0.021529 |
| **IL-1ra** | 0.104839 | **0.354246*** | -0.093496 | -0.177104 | 0.001691 | 0.008165 |
| **IL-2** | **0.227098*** | -0.064881 | **0.331509*** | -0.005912 | 0.001079 | 0.040899 |
| **IL-3** | **0.245752*** | -0.081412 | -0.208912 | -0.000448 | 0.062445 | 0.131445 |
| **IL-4** | **0.243064*** | -0.105143 | -0.199564 | -0.002314 | -0.008982 | 0.126853 |
| **IL-5** | 0.180412 | -0.103294 | -0.23868 | -0.017105 | 0.129446 | -0.206312 |
| **IL-6** | 0.03008 | **0.464398*** | 0.009032 | -0.220458 | 0.02929 | -0.011207 |
| **IL-7** | **0.234645*** | 0.019916 | -0.191298 | -0.047539 | 0.10378 | 0.054054 |
| **IL-8** | 0.102454 | **0.439532*** | 0.052544 | -0.019045 | -0.105226 | 0.01459 |
| **IL-10** | 0.114513 | 0.095138 | -0.092493 | 0.510207 | 0.006453 | **0.350827*** |
| **IL-12 (p40)** | **0.241626*** | -0.05037 | **0.285691*** | -0.050562 | -0.037683 | 0.05385 |
| **IL-12 (p70)** | **0.267594*** | -0.000985 | -0.122491 | -0.045928 | 0.01351 | -0.034343 |
| **IL-13** | **0.230854*** | -0.120057 | -0.249027 | -0.044552 | 0.023699 | -0.001649 |
| **IL-15** | **0.226878*** | -0.024105 | **0.317989*** | 0.054718 | 0.012181 | 0.017692 |
| **IL-17A** | **0.207178*** | 0.024985 | -0.144461 | -0.047302 | -0.070721 | -0.424188 |
| **IP-10** | 0.114513 | 0.095138 | -0.092493 | **0.510207*** | 0.006453 | **0.350827*** |
| **MCP-1** | 0.025349 | **0.263490*** | -0.003862 | **0.557515*** | 0.007126 | -0.130672 |
| **MIP-1α** | 0.093541 | 0.011975 | 0.033128 | 0.001864 | 0.113973 | 0.19125 |
| **MIP-1β** | 0.185627 | 0.116265 | 0.199287 | -0.070006 | 0.122131 | -0.206908 |
| **TNF-α** | **0.243676*** | 0.16158 | -0.113504 | -0.025066 | -0.144477 | 0.1592 |
| **TNF-β** | **0.243156*** | -0.117522 | -0.208143 | -0.028721 | 0.014381 | 0.096658 |
| **VEGF** | 0.173517 | -0.07325 | -0.162853 | -0.028937 | -0.030522 | 0.138279 |

EGF: epidermal growth factor; G-CSF: granulocyte-colony stimulating factor; GM-CSF: granulocyte-macrophage colony-stimulating factor; IFN: interferon; IL: interleukin; IP: inducible protein; MCP: Monocyte Chemo-attractant Protein; MIP: Macrophage inflammatory protein; TNF: tumor necrosis factor; VEGF: vascular endothelial growth factor

^*^ Loading values higher than 0.2.
